# Preparation of g-C_3_N_4_/Co_3_O_4_/NS-CQDs Composite Materials and Their Application in the Detection of Hydrogen Peroxide and Glucose

**DOI:** 10.3390/nano15100752

**Published:** 2025-05-16

**Authors:** Chang Feng, Yufeng Chen, Weie Wang, Yanan Niu, Xi Cao, Yuguang Lv

**Affiliations:** 1 School of Chemistry and Chemical Engineering, Mudanjiang Normal University, Mudanjiang 157000, China; 2 College of Pharmacy, Jiamusi University, Jiamusi 154007, China; 3 School of Art and Design, Qiqihar University, Qiqihar 161006, China; 4 School of Information Science and Technology, Beijing University of Chemical Technology, Beijing 100029, China

**Keywords:** g-C_3_N_4_, peroxidase mimic, N,S-doped carbon quantum dots (NS-CQDs), Co_3_O_4_, colorimetric detection

## Abstract

g-C_3_N_4_, a biocompatible material, has prominent applications in biology and is ideal for nano-enzyme studies. Though reported as a peroxidase mimic, its activity remains low. This group combined N,S-doped carbon quantum dots (NS-CQDs) with g-C_3_N_4_ (7NSC-g), verifying its peroxidase-like activity. Based on this, a ternary composite of Co_3_O_4_ in different forms and 7NSC-g was developed to enhance peroxidase activity, to design a g-C_3_N_4_-based composite enzyme. Characterizations determined the composition and morphology. Colorimetry evaluated peroxidase activity, where the simulated enzyme catalyzes blue product formation from the TMB substrate in the presence of H_2_O_2_. UV-Vis spectrophotometry measured absorbance changes to determine target concentrations. The results show Co_3_O_4_ doping improves catalytic activity, with larger specific surface area providing more activation sites. The highest activity of g-C_3_N_4_/NS-CQDs/Co_3_O_4_ was at 5% floral Co_3_O_4_, being efficient due to Co_3_O_4_’s electron-transfer acceleration and hydroxyl-radical mechanism. Under optimal conditions, the composite detected H_2_O_2_ (10.0–230.0 μM, detection limit of 0.031 μM) and glucose (10.0–650.0 μM, detection limit of 1.024 μM).

## 1. Introduction

Biomolecules usually refer to various types of molecules unique to organisms, whose main function is to regulate, control and maintain the normal function of various organs of the body during the growth and development of biological cells and tissues, and they are related to life activities [1,2]. High levels of H_2_O_2_ attack cell nucleic acids, proteins, etc., causing cell damage and leading to blood vessel damage and cell death. Abnormal levels of glucose in human blood are closely linked to many diseases such as obesity or diabetes. Therefore, monitoring the concentration of H_2_O_2_ and glucose in blood has important practical significance for the prevention and treatment of related diseases [3].

Colorimetric analysis is widely used in the construction of biosensors for small-molecule detection because of its simple, effective, rapid and convenient characteristics [4]. Due to their unique physical and chemical properties, nanomaterials have good application prospects in the detection and sensing of small biological molecules. Some nanomaterials also have catalytic activity similar to that of biological enzymes. These nanomaterials are called nanozymes, and, so far, many nanomaterials with biological-enzyme activity have been found, such as precious-metal nanoparticles, metal-oxide nanoparticles, carbon nanoparticles, etc. In carbon-based nanomaterials, studies have shown that g-C_3_N_4_ has low peroxidase-like catalytic activity [5], so further modification is needed to prepare more efficient peroxidase-like mimics. At the same time, studies have shown that N-doped carbon-based materials can change the electron arrangement of CQDs [6], increase the electron-giving ability and produce active sites with high catalytic activity, while S doping can change the surface groups and conjugated systems of CQDs [7], thereby improving the optical properties of CQDs [8,9]. At the same time, many metals and metal oxide materials have been explored for POD catalysis. In order to achieve a significant increase in the efficiency of the catalytic cycle, a new type of active metal center is essential. Due to the similar properties of Co and Fe, it should have variable valences, and high-priced ions must be reduced more quickly. The Co compound was used instead of the Fe compound in the catalytic reaction of H_2_O_2_ and showed excellent catalytic activity for a long time [10,11]. Therefore, based on previous studies, a Co_3_O_4_/NSC-g ternary composite was designed and synthesized for the first time in this experiment, and the influence of different Co_3_O_4_ forms and different Co_3_O_4_ doping amounts on enzyme activity was investigated to study its catalytic effect [12].

## 2. Materials and Methods

### 2.1. Materials

Urea (99.9%), L-cysteine (99.9%), NaOH (97%), 1,2-dimethylimida-zole (98%) and Co(NO_3_)_2_·6H_2_O (AR,99%) were bought from Aladdin Holdings Group (PRC). NaNO_3_ (99%), NH_3_·H_2_O (GR, 25–28%) and H_2_O_2_ (AR, 30 wt.% in H_2_O) were purchased from Sinopharm Chemical Reagent Co., Ltd. (Shanghai, China)Methanol (AR, 99.5%) and anhydrous ethanol (AR, 99.7%) were purchased from Tianjin Kaitong Chemical Reagent Co., Ltd. (Tianjin, China). All the water used in the laboratory was deionized water.

The samples were analyzed by X-ray diffraction (XRD) using a SmartLab (3KW) X diffractometer manufactured by Rigaku Corporation (Japan). This advanced instrument, sourced from Tokyo, Japan with a scanning speed of 0.5°/min and a scanning range of 10 to 90°. An Al K-alpha1063 X-ray photoelectron spectrometer (XPS) manufactured by Thermo Fisher Scientific was chosen to analyze the chemical composition and elemental valence states of the photocatalysts. It sourced from Horsham, United Kingdom, utilizes Al Kα X-rays were used as the light source, with an accelerating voltage of 12 kV, an energy voltage of 1500 eV, and an energy scanning step of 0.05 eV. For the calibration of the binding energy (BE), the C 1s peak of adventitious carbon at 284.8 eV was used as a reference. During the XPS measurements, a pass energy of 150 eV was employed for the Survey spectra to obtain a quick overview of the elemental composition. For the high-resolution spectra, a lower pass energy of 20 eV was used to achieve higher energy resolution for detailed analysis of the chemical states of the elements. A UV-2700 UV-vis spectrophotometer manufactured by Shimadzu Corporation was chosen to analyze the absorption spectra (UV-vis DRS) of the photocatalyst powders, which were tested using BaSO4 as a reference, with a scanning band range of 200–800 nm and a slit of 5 nm. This device is from Kyoto, Japan. FTIR spectra with absorbance values expressed in cm^−1^ were recorded using a PerkinElmer RX-I FTIR spectrometer by PerkinElmer, Inc. This device is from Bikensfield, UK. The samples were characterized by scanning electron microscopy (SEM JMS-7900F) manufactured by JEOL Ltd. (Japan), and by using a JEM-2010 transmission electron microscope equipped with EDX at a voltage of 200 kV and a current of 101 mA, with a maximum accelerating voltage of 200 kV and a maximum α tilt angle of 45°. This scanning electron microscope originated in Tokyo, Japan.

### 2.2. Preparation of Co_3_O_4_/NSC-g Composites with Different Morphologies

The preparation of NS-CQDs/g-C_3_N_4_: 0.2 g of g-C_3_N_4_ was weighed and dispersed into 30.0 mL of anhydrous ethanol. A certain amount of aqueous NS-CQDs was added, and the mixture was ultrasonically stirred for 1 h. The reaction was heated in a high-pressure reactor at 120 °C for 2 h. The samples were cooled, collected by centrifugation and washed three times each with anhydrous ethanol and distilled water. Finally, the samples were dried at 60 °C for 12 h. The obtained NS-CQDs/g-C_3_N_4_ nanosheet composites were denoted as NSC-g.

Preparation of spherical Co_3_O_4_/NSC-g composites: 3.65 g of Co(NO_3_)_2_ and 0.85 g of NaNO_3_ were dissolved in 40.0 mL of deionized water. After complete dissolution, 20.0 mL of ammonia was added, and the solution was magnetically stirred for 15 min until the color stabilized. Then, 4.0 mL of H_2_O_2_ was added dropwise, and the mixture was stirred for another 30 min. The solution was transferred to an autoclave reactor and heated at 120 °C for 12 h. After cooling to room temperature, the products were separated, washed several times with deionized water and dried to obtain black Co_3_O_4_. NSC-g was mixed with spherical Co_3_O_4_ at mass ratios of 1%, 3%, 5%, 7% and 10%. The mixtures were ground thoroughly and calcined in a muffle furnace at 400 °C for 3 h. Spherical Co_3_O_4_/NSC-g composites with different doping ratios were obtained and labeled as spherical X% Co_3_O_4_/7NSC-g (X = 1, 3, 5, 7, 10).

Preparation of flower-like Co_3_O_4_/NSC-g composites [13,14]: 1.97 g of 1,2-dimethylimidazole and 1.746 g of Co(NO_3_)_2_·6H_2_O were weighed and dissolved in 20.0 mL of methanol and 20.0 mL of anhydrous ethanol, respectively, and stirred at 20 °C for 48 h. The precipitates were separated and washed with anhydrous ethanol to remove unreacted 1,2-dimethylimidazole and Co^2+^. The precipitate was washed with anhydrous ethanol to remove unreacted 1,2-dimethylimidazole and Co^2+^. The precipitate was dried at 50 °C to obtain flower-like Co_3_O_4_, and the NSC-g and flower-like Co_3_O_4_ powders were ground in the ratio of 1%, 3%, 5%, 7%, 10% by mass, respectively, and placed in a muffle furnace and calcined at 350 °C for 2 h to obtain the flower-like Co_3_O_4_/7NSC-g materials with different composite ratios. The samples were labeled as flower-like X% Co_3_O_4_/7NSC-g according to the composite ratio (X = 1, 3, 5, 7, 10).

### 2.3. POD-like Activity Analysis

Briefly, 100 μL of 1 mg/mL Co_3_O_4_/7NSC-g dispersion, 120 μL of 5 mM H_2_O_2_ and 100 μL of 8 mM TMB were sequentially added to 0.2 M NaAc-HAc buffer at pH 4.0. The reaction mixture was thoroughly mixed, and the kinetic spectra were recorded over 300 s using an ultraviolet spectrophotometer. For each control group, the system was incubated in a 35 °C water bath for 20 min before scanning the UV absorption spectra in the 550–750 nm range.

### 2.4. Steady-State Kinetic Study

The steady-state kinetics of the flower 5% Co_3_O_4_/7NSC-g composites were investigated using Michaelis–Menten kinetic analysis. For H_2_O_2_ as the variable substrate, 100.0 μL of flower-like 5% Co_3_O_4_/7NSC-g and 100.0 μL of TMB (fixed at 0.1 mM) were added to 2680.0 μL of buffer solution. Then, 120.0 μL of H_2_O_2_ solutions with different concentrations were introduced, and the kinetic spectra were recorded at 652 nm over 300 s after mixing. All the other component concentrations were kept constant. For TMB as the variable substrate, the H_2_O_2_ concentration was fixed at 5 mM, and various concentrations of TMB were added. The kinetic spectra were scanned at 652 nm for 300 s after mixing. We have placed reaction mechanism studies, hydrogen peroxide and glucose assays in the Supplementary Materials.

## 3. Results and Discussion

### 3.1. Characterization of Co_3_O_4_/NSC-g Composites

**Figure 1:**
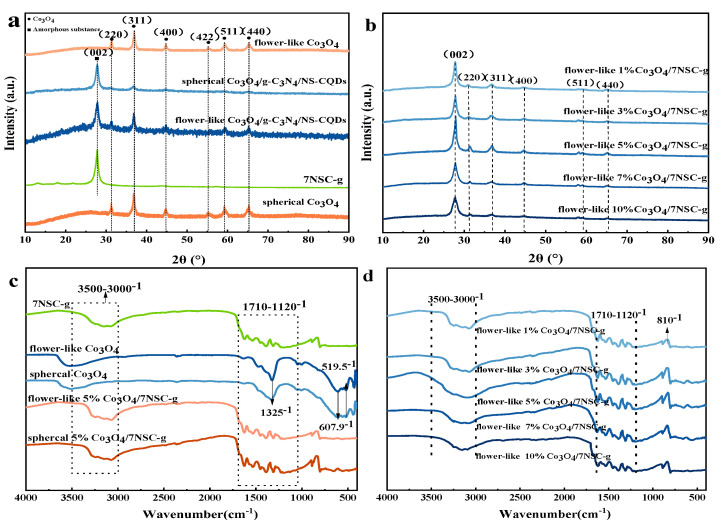
(**a**,**b**) XRD and (**c**,**d**) F−IR spectra of different composites.

The XRD spectra (Figure 1a,b) of different composites exhibited diffraction peaks at 31.4°, 36.8°, 44.8°, 55.4°, 59.4°, and 65.3°, corresponding to the (220), (311), (400), (422), (511), and (440) planes of Co_3_O_4_, as well as a diffraction peak at 27.4°, which corresponded to the (002) plane of g-C_3_N_4_ [15]. Compared with those of Co_3_O_4_, the above peaks of the flower-like Co_3_O_4_/NSC-g composites showed different degrees of intensity reduction or broadening, which indicates the agglomeration of Co_3_O_4_ in the flower-like Co_3_O_4_/NSC-g composites [16,17,18]. It is worth noting that, when the doping amount of flower-like Co_3_O_4_ was 5%, the characteristic peaks in the graphs were sharper and more intense compared with those for other doping amounts, which indicates better crystallinity.

Analyzing the FT-IR spectra provides functional group and chemical bonding information of the samples [19]. As shown in Figure 1c, the stretching vibration peaks of Co_3_O_4_ with different morphologies at 607.9 cm^−1^ and 519.5 cm^−1^ correspond to Co^2+^-O and Co^3+^-O, respectively. The broad peaks at 1325 cm⁻¹ and 3527 cm⁻¹ are attributed to the bending and stretching vibrations of O-H bonds from physically adsorbed water [20,21]. From Figure 1d, C-N stretching vibrations in the aromatic ring (1120–1710 cm^−1^) are retained in Co_3_O_4_/NSC-g composites with different Co_3_O_4_ doping amounts. The bending vibration peak of the triazine ring—the basic structural unit of g-C_3_N_4_—at 810 cm⁻¹ confirms the presence of g-C_3_N_4_ in the composites. Notably, with an increasing Co_3_O_4_ loading, the triazine ring characteristic peak shifted from 810 cm^−1^ to 803 cm^−1^ in Co_3_O_4_/NSC-g, indicating weakened triazine ring strength in g-C_3_N_4_. This may result from the effect of Co_3_O_4_ introduction on the bonding energy of NSC-g [22].

**Figure 2:**
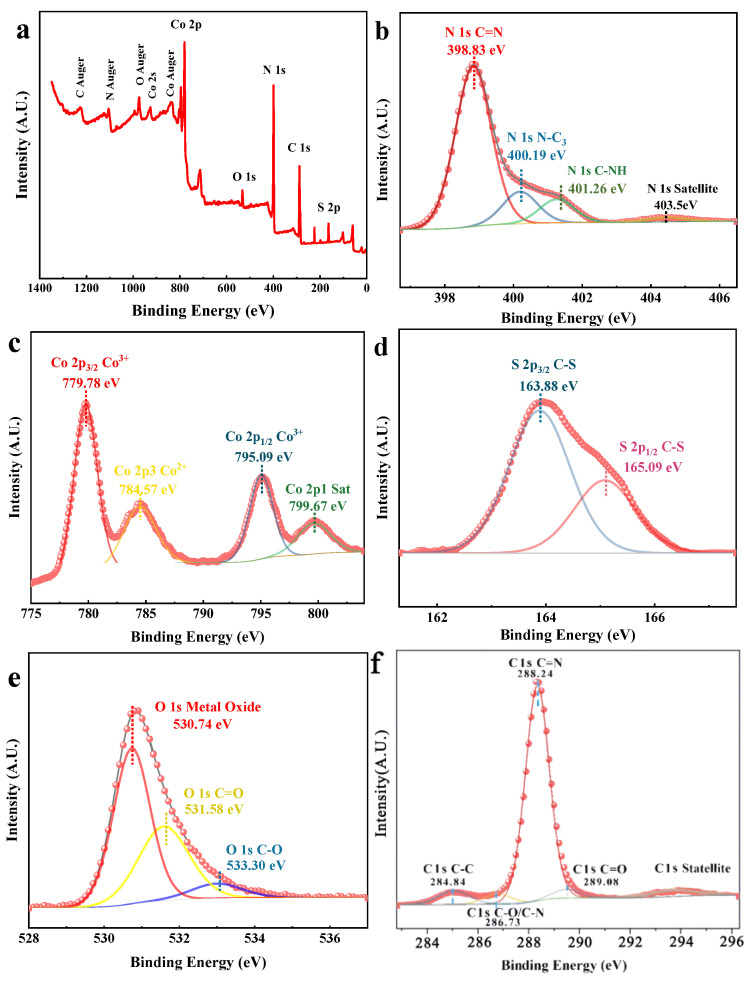
XPS energy spectra of Co_3_O_4_/NSC-g in (**a**) full spectrum, (**b**) N 1 s, (**c**) Co 2p, (**d**) S 2p, (**e**) O 1 s and (**f**) C 1 s.

The XPS spectra of Co_3_O_4_/NSC-g were measured. The full spectrum is shown in Figure 2a, which indicates that the composite consists of five elements: N, C, O, S and Co [23]. The high-resolution spectrum of N 1 s (Figure 2b) shows three distinct peaks at 398.83 eV, 400.19 eV and 401.26 eV, which correspond to the sp^2^-bonded N-C=N, N-C_3_ and C-NH (amino) in the triazine ring, respectively [24]. Figure 2c shows the Co 2p spectra. The peak at 779.78 eV corresponds to the characteristic peak of Co 2p_3/2_. After peak-splitting fitting, it was found that the peak at 779.78 eV corresponds to Co^3+^ in Co_3_O_4_, and the peak at 784.57 eV corresponds to Co^2+^. The peak at 795.09 eV is the characteristic peak of Co 2p_1/2_. After peak-splitting fitting, the binding energy at 795.09 eV corresponds to Co^3+^ in Co_3_O_4_, and the peak at 799.67 eV corresponds to Co^2+^. This proves the presence of Co^3+^ and Co^2+^ in the prepared composite [25]. The S 2p spectra (Figure 2d) have peaks at binding energies of 163.88 eV and 165.09 eV, corresponding to S 2p_3/2_ and S 2p_1/2_, respectively. The O 1 s spectrum (Figure 2e) shows that the peak at 530.74 eV corresponds to Co-O bonds in Co_3_O_4_ (both Co^2^⁺ and Co^3+^), and the peaks at 531.58 eV and 533.3 eV correspond to C=O and C-O bonds. The high-resolution spectrum of C 1 s (Figure 2f) does not change much compared with that of C 1 s of NSC-g [26]. Although the positions of the characteristic peaks change slightly, the corresponding bonding patterns remain the same. The peaks at 284.84 eV, 288.24 eV and 289.08 eV correspond to C=C, N-C=N and C-O, respectively [27].

**Figure 3:**
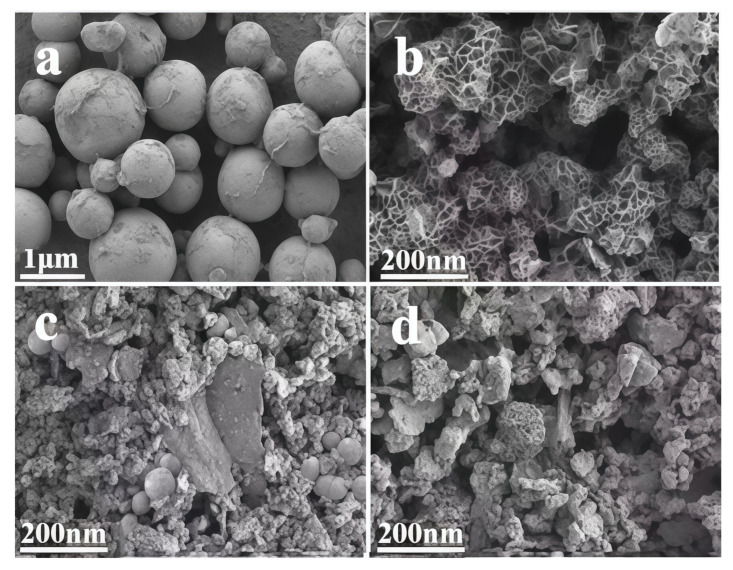
SEM images of (**a**) spherical Co_3_O_4_, (**b**) flower-like Co_3_O_4_, (**c**) spherical 5% Co_3_O_4_/NSC-g and (**d**) flower-like 5% Co_3_O_4_/NSC-g.

In Figure 3a, uniform Co_3_O_4_ nanospheres are observed, while Figure 3b clearly shows flower-like Co_3_O_4_ with a hierarchical structure composed of multiple layers of ultrathin nanosheets. Figure 3c and d reveal that both spherical and flower-like Co_3_O_4_ are uniformly dispersed in the NSC-g matrix. Comparison indicates that flower-like Co_3_O_4_ has a larger specific surface area, enabling more substrate adsorption and providing additional active sites for the catalytic reaction [28].

**Figure 4:**
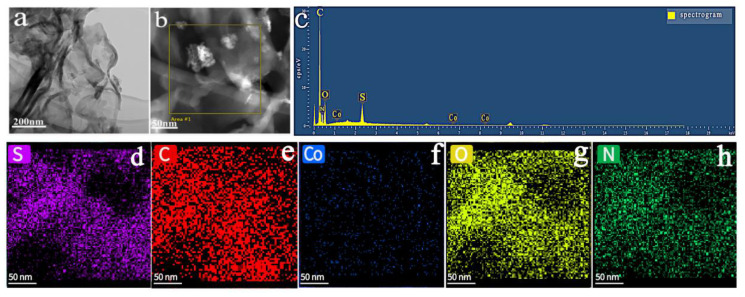
(**a**,**b**) TEM image of flower-like Co_3_O_4_, elemental mapping images of (**c**) EDX spectrum, (**d**) S-element, (**e**) C-element, (**f**) Co-element, (**g**) O-element, (**h**) N-element.

From the TEM images, the prepared flower 5% Co_3_O_4_/7NSC-g is observed to retain a lamellar structure with transparent regions (Figure 4a), indicating that the composite’s matrix remains an ultrathin g-C_3_N_4_ nanosheet. The presence of flower-like Co_3_O_4_ nanoparticles is evident in Figure 4b. The chemical composition of the synthetic material was estimated by EDX analysis and found to contain the elements C, N, O, S, and Co, as shown in Fig. 4c. Elemental mapping (Figure 4d–h) revealed uniform distributions of S, C, Co, Os and N across the composite, confirming the successful loading of flower-like Co_3_O_4_ onto the NSC-g surface [29].

### 3.2. POD-like Activity Studies of Co_3_O_4_/NSC-g Composites

#### 3.2.1. Validation of POD-like Activity

**Figure 5:**
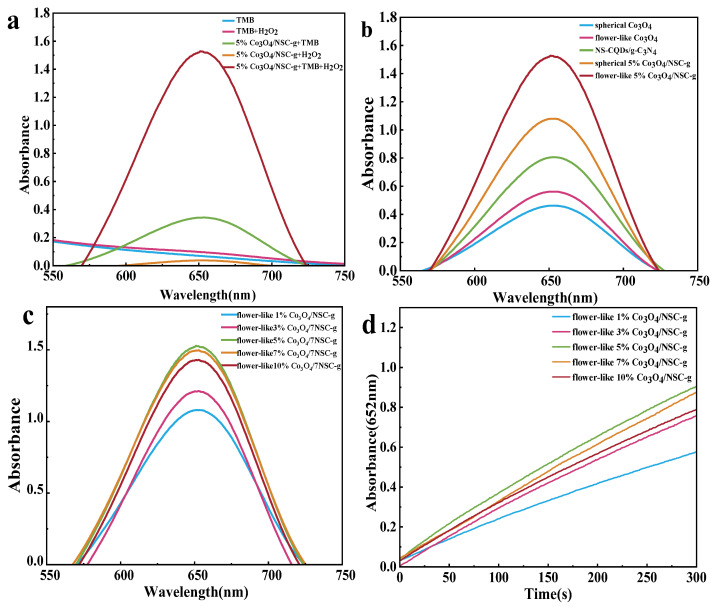
(**a**) Ultraviolet–visible absorption spectra of different reaction systems; (**b**) ultraviolet–visible absorption spectra of different peroxidase material reaction systems; (**c**) UV-Vis absorption spectra of NSC-g with different doping amounts of Co_3_O_4_ and (**d**) its time–kinetic curves (λ = 654 nm).

To investigate the peroxidase-like activity of flower-like (or spherical) X% Co_3_O_4_/NSC-g composites, an X% Co_3_O_4_/NSC-g-TMB-H_2_O_2_ system was constructed. Based on the characteristic absorption peak of oxidized TMB (ox TMB) at λ = 652 nm during TMB oxidation, the system produced no or weak absorption peaks when lacking H_2_O_2_, TMB, or flower-like X% Co_3_O_4_/NSC-g. Notably, as shown in Figure 5a, a weak absorption peak still emerged in the absence of H_2_O_2_, indicating that flower-like 5% Co_3_O_4_/NSC-g exhibits trace oxidase-like activity [30].

The peroxidase-like activity results were compared using different catalytic materials in the system (Figure 5b), indicating that Co_3_O_4_ introduction significantly enhanced the catalytic performance of 7NSC-g. The peroxidase-like activities of composites with varying flower-like Co_3_O_4_ loadings were further compared, and their kinetic profiles were analyzed. As shown in Figure 5c,d, the optimal catalytic activity occurred at a 5% flower-like Co_3_O_4_ loading, which was selected for subsequent experiments.

#### 3.2.2. Steady-State Kinetic Studies

**Figure 6:**
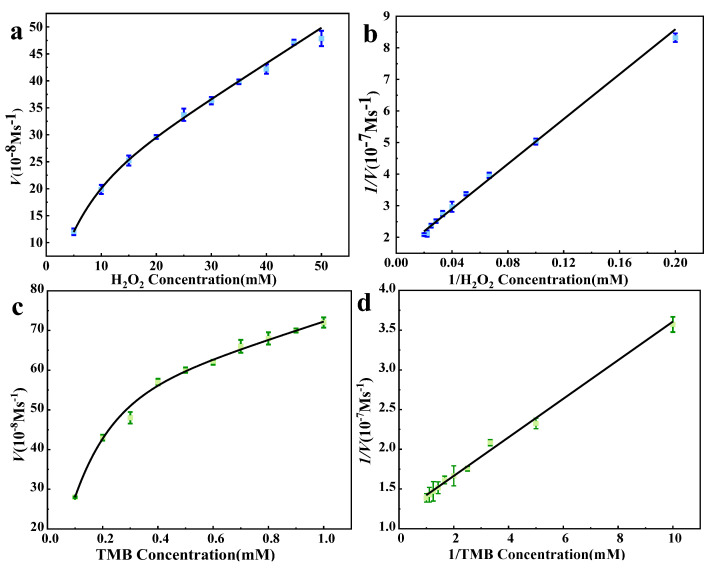
The steady−state kinetic curves of flower-like 5% Co_3_O_4_/NSC−g nanase; the kinetic curves of H_2_O_2_ (**a**) and the double reciprocal curve (**b**) at a fixed TMB concentration of 0.1 mM; kinetic curve (**c**) and double reciprocal curve (**d**) of TMB at fixed H_2_O_2_ concentration of 5 mM.

To investigate the catalytic mechanism of 5% Co_3_O_4_/7NSC-g, the catalytic kinetic process was studied. As shown in Figure 6, it is the steady-state kinetic curve of the flower-shaped 5% Co_3_O_4_/NSC-g enzyme. Km and Vmax were obtained by fitting the Lineweaver–Burk double-reciprocal curve to the Michaelis–Menten equation. A smaller Km value indicates stronger enzyme–substrate affinity. The kinetic parameters are shown in Table 1. Comparing the Km values of the two substrates, flower-like 5% Co_3_O_4_/7NSC-g nanozymes exhibited stronger affinity for the TMB substrate. This enhanced affinity can be attributed to two factors: (1) the flower-like 5% Co_3_O_4_/7NSC-g nanozymes feature a hierarchical porous structure and large specific surface area, which facilitate substrate adsorption and provide abundant active sites; (2) Co^3+^ in Co_3_O_4_ can directly oxidize TMB via electron transfer, leading to stronger TMB affinity.

### 3.3. The Basic Mechanism of the Reaction

#### Determination of Free Radicals

**Figure 7:**
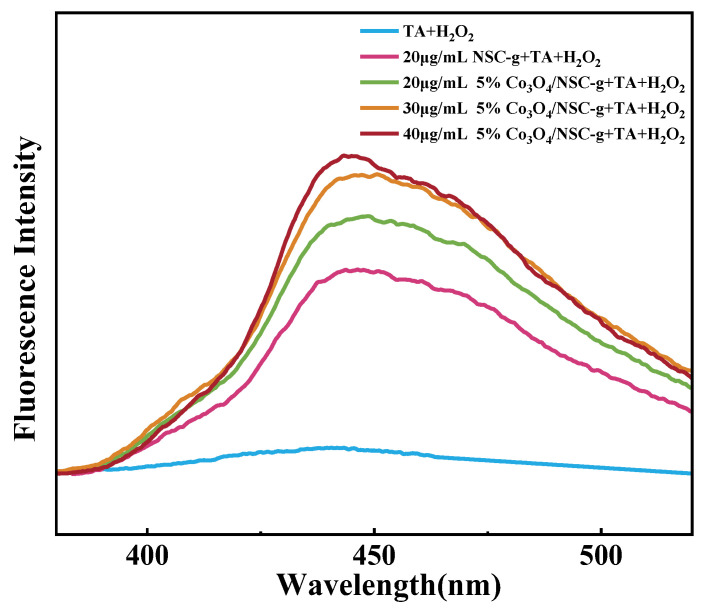
Fluorescence spectra of different reaction systems added to TA + H_2_O_2_.

TA itself is non-fluorescent, but it can generate fluorescent 2-hydroxyterephthalic acid upon capturing ·OH in the reaction system [21]. As shown in Figure 7, the fluorescence intensity of the flower-like 5% Co_3_O_4_/7NSC-g nanocomposite is slightly higher than that of 7NSC-g, but the increase is minimal. This suggests that Co_3_O_4_ nanoparticles and H_2_O_2_ do not significantly enhance ·OH production, and the improved performance primarily stems from the increased specific surface area and active sites. The high peroxidase activity of 5% Co_3_O_4_/7NSC-g originates not only from Co_3_O_4_’s excellent conductivity, which accelerates electron transfer during the reaction, but also from its ability to catalytically generate more ·OH from H_2_O_2_.

### 3.4. Detection of Hydrogen Peroxide and Actual Samples

#### 3.4.1. Detection of Hydrogen Peroxide

**Figure 8:**
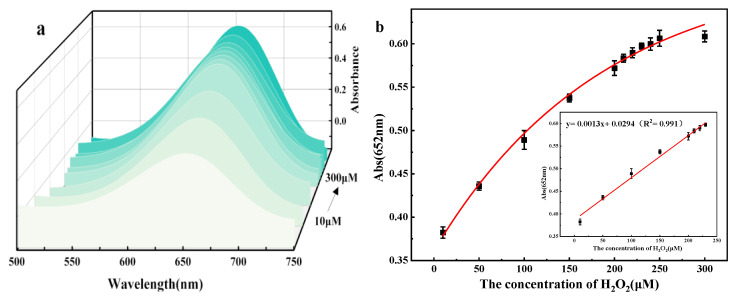
(**a**) UV-vis absorption spectrum with H_2_O_2_ concentration; (**b**) the concentration change curve of H_2_O_2_ detection and the corresponding standard curve of H_2_O_2_ detection.

Under optimal experimental conditions, the UV-Vis spectra of the TMB + H_2_O_2_+ flower-like 5% Co_3_O_4_/NSC-g reaction system with different H_2_O_2_ concentration gradients were scanned using a UV-Vis spectrophotometer. Figure 8a shows the UV-Vis absorption spectra at different H_2_O_2_ concentrations. Figure 8b shows that the absorbance at λ = 652 nm increased with the H_2_O_2_ concentration in the solution. There was a linear relationship between the absorbance and the H_2_O_2_ concentration in the range of 10.0–230.0 μM, expressed as A = 0.0013 C_(H2O2)_ + 0.0294 (R^2^ = 0.991), and the detection limit was 0.031%.

#### 3.4.2. Detection of Hydrogen Peroxide in Milk

Based on the excellent performance of flower-like 5% Co_3_O_4_/NSC-g in H_2_O_2_ detection, it was applied to determine H_2_O_2_ in real milk samples. The accuracy and precision were evaluated using the standard addition method [26]. Spiked milk samples were prepared by adding H_2_O_2_ at concentrations of 50.0, 100.0 and 150.0 μM to diluted milk. The recoveries ranged from 98.46% to 101.74%, with relative standard deviations (RSDs) ≤ 3.62%, indicating the method’s reliability for real-sample analysis (Table 2).

### 3.5. Detection of Glucose and Actual Samples

#### 3.5.1. Detection of Glucose

**Figure 9:**
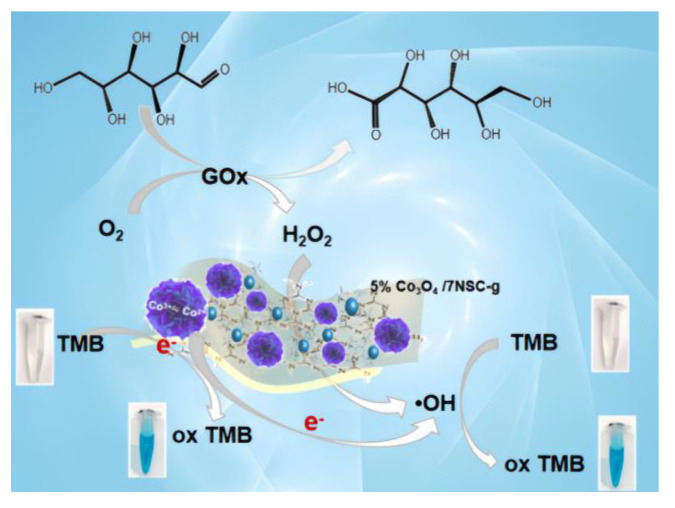
Mechanism diagram of glucose detection by flower-like 5%Co_3_O_4_/NSC-g.

GOx catalyzes the oxidation of glucose to gluconic acid and H_2_O_2_, enabling indirect glucose detection via absorbance measurement at λ = 652 nm [30]. The detection principle is illustrated in Figure 9, where colorimetric glucose determination is achieved based on absorbance changes in the reaction system.

**Figure 10:**
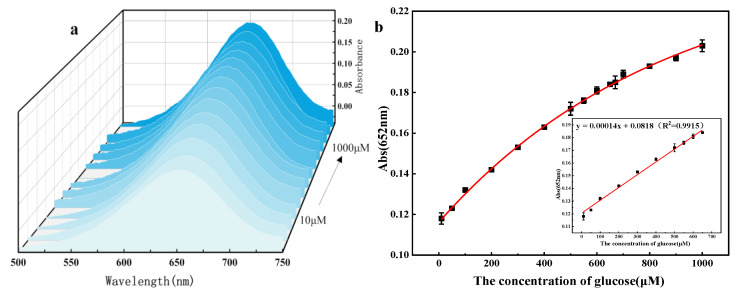
(**a**) UV-vis absorption spectrum with glucose concentration, (**b**) the curve of the glucose detection concentration change and the corresponding standard curve of glucose detection.

Based on the above reaction mechanism, a colorimetric glucose detection system was constructed using flower-like 5% Co_3_O_4_/NSC-g nanozymes as a platform under optimal experimental conditions. The UV absorption spectra of different glucose concentrations are shown in Figure 10a. As demonstrated in Figure 10b, the absorbance at λ = 652 nm increased with glucose concentration, exhibiting a good linear relationship in the 10.0–650.0 μM range. The linear equation is expressed as follows: A = 0.00014C_₍glucose₎_ + 0.0818 (R^2^ = 0.9915) with a detection limit of 1.024 μM.

#### 3.5.2. Detection of Glucose in Urine

To evaluate the feasibility of flower-like 5% Co_3_O_4_/NSC-g for real-sample detection, glucose concentrations in spiked urine samples were measured after adding glucose to diluted simulated urine. The recoveries ranged from 98.46% to 104.5%, with relative standard deviations (RSDs) ≤3.75%, as shown in Table 3. This indicates the colorimetric method based on this nanozyme exhibits high reliability for glucose detection in real urine samples.

## 4. Conclusions

In this study, Co_3_O_4_/7NSC-g composites with different shapes were prepared to investigate the peroxidase-like activity of the composites. The effects of different Co_3_O_4_ doping on the enzyme activity of the composites were discussed. The composites exhibited maximum catalytic activity when the flower-like Co_3_O_4_ doping was 5%. Their efficient catalytic ability originated from the introduction of Co_3_O_4_, which accelerates electron transfer and follows the hydroxyl radical mechanism. The steady-state kinetics is consistent with the Michaelis–Menten model, which is due to the fact that, on the one hand, the main-material g-C_3_N_4_ nanosheets of the flower 5% Co_3_O_4_/NSC-g has rich void structure and large surface area, which is conducive to the adsorption of more substrates and more active sites, and, on the other hand, the Co^3+^ in the Co_3_O_4_ is able to oxidize the TMB directly through the electron transfer, and the affinity to the TMB is also high. On the other hand, Co^3+^ of Co_3_O_4_ can directly oxidize TMB through electron transfer, which has a stronger affinity for TMB. Under the optimal experimental conditions, the floral-like 5% Co_3_O_4_/7NSC-g was applied to the detection of hydrogen peroxide in milk samples with a good linear presence and detection limit of 0.031 μM in the range of 10~230 μM. When used for the detection of glucose, it showed a good linear relationship within 10~650 μM, with a detection limit of 1.024 μM, which is lower than the detection limit of similar materials, and had good selectivity and practicability.

## Figures and Tables

**Table 1:** K_m_ and V_max_ comparison of nanozymes.

Nanozymes	H_2_O_2_		TMB	
	K_m_ (mM)	V_max_ (10^−8^ M·S^−1^)	K_m_ (mM)	V_max_ (10^−8^ M·S^−1^)
NSC-g	1.55	4.47	0.170	4.81
Flower-like 5%Co3O4/NSC-g	1.538	6.17	0.1164	5.78

**Table 2:** The content of H_2_O_2_ in milk was determined.

Added H_2_O_2_ (μM)	Found H_2_O_2_ (μM)	Recovered (%)	RSD (%)
50.0	50.48	100.96	1.08
100.0	98.46	98.46	3.28
150.0	152.62	101.74	3.62

**Table 3:** The amount of glucose in urine was measured.

Added Glucose (μM)	Found Glucose (μM)	Recovered (%)	RSD (%)
50.0	50.48	100.96	1.08
100.0	98.46	98.46	3.28
150.0	152.62	101.74	3.62

## Data Availability

These data are available upon request.

## Supplementary Materials

The following supporting information can be downloaded at: https://www.mdpi.com/article/10.3390/nano15100752/s1.
