# Supplementary material for: Preparation of g-C3N4/Co3O4/NS-CQDs Composite Materials and Their Application in the Detection of Hydrogen Peroxide and Glucose
Source: Nanomaterials (Basel). 2025 May 16;15(10):752. doi: 10.3390/nano15100752 (PMC12113748; doi:10.3390/nano15100752)
Supplement: Supplementary file 1 [file nanomaterials-15-00752-s001.zip › nanomaterials-3519602-supplementary.pdf]

2.5 Study of the basic mechanism of the reaction

Terephthalic acid (TA) was used as a fluorescent probe for ·OH to investigate whether the mechanism of fiower 5%Co_3O_4/7NSC-g as a POD mimetic enzyme is associated with ·OH production. After 100.0 μL of 0.6 mM TA was added to 0.2 M NaAc-HAc buffer system with pH=4.0, 100.0 μL of different concentrations of fiower 5% Co_3O_4/7NSC-g dispersion and 120.0 μL of 5 mM H_2O_2 were added sequentially to make the total volume of the mixture 3.0 mL, and then the mixture was incubated at 37 °C for 6 h. The fluorescence spectra were scanned in the range of 325 nm to 600 nm. The fluorescence spectra were scanned in the range of 325 nm-600 nm (λ_{ex}=315 nm), and the corresponding substances were not added in the control experiments, and each group of experiments was measured three times in parallel[15].

2.6 Detection of hydrogen peroxide and hydrogen peroxide in milk

100.0 μL of 1 mg/mL of flower-like 5% Co_3O_4/7NSC-g and 100.0 μL of 8 mM TMB were mixed, and then 120.0 μL of different concentrations of H_2O_2 and 0.2 M NaAc-HAc buffer with pH=4.0 were added to the total volume of 3.0 mL, and the reaction was carried out for 20 min by shaking the solution well, and then the reaction was heated up to 35°C in a water bath and measured by UV-vis spectrophotometer. The reaction was carried out for 20 min and the absorbance at 652 nm was measured by UV-vis spectrophotometer.

A 0.2 M NaAc-HAc buffer solution (pH=4.0) was added to the commercially available boxed milk to precipitate it, and the supernatant was taken after centrifugation for several times and further diluted by adding deionized water to remove the interference of impurities. Different concentrations of H_2O_2 were prepared and added to the diluted milk samples to obtain spiked milk. The steps are the same as above except that 120 uL of milk with different concentrations is used instead of hydrogen peroxide.

2.7 Detection of glucose and glucose in urine

Different concentrations of glucose solution (10.0-350.0 uM) were prepared and mixed with 1 mg/mL GOx, heated at 37°C for 30 min, 120.0 uL of the mixed solution was aspirated and added with appropriate amount of 0.2M NaAc-HAc buffer solution at pH=4.0, 100.0 uL of 8 mM TMB solution, 100.0 uL of 1mg/mL fiower 5% Co_3O_4/7NSC-g dispersion to make a total volume of 3.0 mL, and heated at 35°C for 20 min. Co_3O_4/7NSC-g dispersion to make the total volume of

the system is 3.0 mL, 35 °C water bath heating for 20 min, and then UV-vis spectrophotometer three times in parallel to determine the absorbance of the reaction system at 652 nm.

The purchased simulated human urine was centrifuged several times to remove impurities, and then diluted by adding an appropriate amount of 0.2 M NaAc-HAc buffer solution at pH=4.0. 200.0 uL of diluted urine was accurately aspirated, and 100.0 uL of different concentrations of standard glucose solution and 1 mg/mL of GOx were added to the sample, and the sample was then heated in a water bath at 37 °C for 30 min to obtain the diluted and spiked sample. The diluted and spiked urine samples were obtained after heating at 37 °C for 30 min. Except that 120.0 uL of the labeled urine sample was used to replace the glucose-glucose oxidase mixture, the other steps of the assay were the same as the above steps.
